# IgG4-Related Cholangitis

**DOI:** 10.1055/a-2588-3875

**Published:** 2025-05-08

**Authors:** Ulrich Beuers, David C. Trampert

**Affiliations:** 1 Department of Gastroenterology and Hepatology, Tytgat Institute for Liver and Intestinal Research, AGEM, Amsterdam University Medical Center, Amsterdam, The Netherlands

**Keywords:** autoimmune pancreatitis, biliary bicarbonate umbrella, cholangiocarcinoma, IgG4-related disease, primary sclerosing cholangitis

## Abstract

IgG4-related cholangitis (IRC) is a rare fibroinflammatory disease of the biliary tree and liver and presents the major hepatobiliary manifestation of IgG4-related systemic disease (IgG4-RD). IRC also includes the IgG4-related inflammatory pseudotumor of the liver and IgG4-related cholecystitis. IRC mimics other cholangiopathies such as primary sclerosing cholangitis or cholangiocarcinoma. IRC may be found in 30 to 60% of cases with type 1 autoimmune pancreatitis, the most frequent manifestation of IgG4-RD. The pathogenesis of IRC (and IgG4-RD) is incompletely understood. Genetic predisposition, environmental factors, oligoclonal glucocorticosteroid-sensitive expansion of IgG4 ^+^ B cells/plasmablasts in blood and affected tissue and blocking autoantibody formation against protective IgG4-specific autoantigens such as annexin A11 and laminin 511-E8 with impaired protection of biliary epithelia against toxic bile acids have been described in IRC. Specific T cell subtypes are involved in the inflammatory process. The diagnosis of IRC is made according to HISORt criteria comprising histopathology, imaging, serology, other organ manifestations, and response to therapy. Treatment of IRC aiming to prevent organ failure and improve symptoms includes remission induction with highly effective glucocorticosteroids and long-term maintenance of remission with immunomodulators such as glucocorticosteroid sparing additives or B cell depleting approaches.

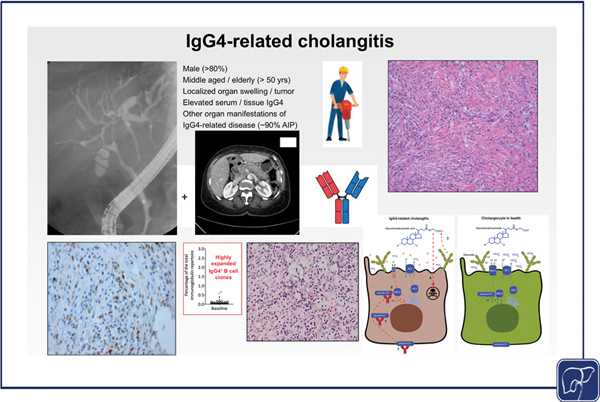

IgG4-related cholangitis (IRC), 1 also known as IgG4-related cholangiopathy, is the major hepatobiliary manifestation of IgG4-related disease (IgG4-RD), 2 3 a rare systemic fibroinflammatory disorder of yet unknown pathogenesis which can predominantly affect secretory organs, 4 but also other sites in the human body. IgG4-related inflammatory pseudotumors predominantly in the perihilar region of the liver are regarded as part of IRC as is IgG4-related cholecystitis. An IgG4-related hepatopathy with parenchymal histopathological features reminding of sequelae to obstructive cholestasis as a possible consequence of IRC and lacking the mandatory histological criteria for IgG4-RD, “storiform fibrosis” and “obliterative phlebitis,” 5 has also been discussed. 6 Finally, the incompletely explored and not yet established concept of an “IgG4-related autoimmune hepatitis” has been introduced and controversially discussed as a rare hepatobiliary manifestation of IgG4-RD 6 7 8 where it is difficult to completely exclude, for example, a toxin-induced autoimmune-like hepatitis in genetically predisposed individuals which later may show other features of IgG4-RD. 6 7 Meanwhile, numerous organ manifestations of IgG4-RD have been reported ( Table 1 ) 2 and their numbers are increasing, not in all cases following consensus criteria. The present review focuses on IRC, the major hepatobiliary manifestation of IgG4-RD, and is an update of a recent extended review by our group on IRC 1 ; therefore, major overlap cannot be avoided.

**Table 1:** Organs that can be affected by IgG4-RD and disease manifestations (incomplete)

Organ	Manifestation of IgG4-RD
Head and neck	
Pachymeninges	IgG4-related pachymeningitis
Hypophysis	IgG4-related hypophysitis
Eyes	IgG4-related ophthalmic/orbital disease
Lacrimal glands	IgG4-related dacryoadenitis (Mikulicz's syndrome)
Salivary glands –Parotid glands –Submandibular glands	IgG4-related sialadenitis (Mikulicz's syndrome) IgG4-related parotitis IgG4-related submandibular gland disease (Küttner's tumor)
Thyroid gland	IgG4-related thyroiditis (Riedel's struma)
Lymph nodes	IgG4-related lymphadenopathy
Thorax	
Lung	IgG4-related lung disease
Mediastinum	IgG4-related mediastinitis
Lymph nodes	IgG4-related lymphadenopathy
Pericardium	IgG4-related pericarditis
Pleura	IgG4-related pleuritis
Mamma	IgG4-related mastitis
Abdomen	
Pancreas	Type 1 autoimmune pancreatitis –With/without inflammatory pseudotumors
Liver and bile ducts, gallbladder	IgG4-related cholangitis –With/without inflammatory pseudotumors IgG4-related cholecystitis IgG4-related hepatopathy (possibly secondary to IRC) IgG4-related autoimmune hepatitis?
Stomach	IgG4-related gastric disease
Intestine	IgG4-related intestinal disease
Mesentery	IgG4-related mesenteritis
Kidney Tubuli Glomeruli Pyelum Ureters	IgG4-related kidney disease –Tubulointerstitial nephritis –Membranous glomerulonephritis –Renal pyelitis –IgG4-related ureteritis (with/without retroperitoneal fibrosis)
Retroperitoneum	IgG4-related retroperitoneal fibrosis
Aorta	IgG4-related aortitis/periaortitis
Arteries	IgG4-related periarteritis
Lymph nodes	IgG4-related lymphadenopathy
Pelvis	
Prostate	IgG4-related prostatitis
Epididymis	IgG4-related epididymis pseudotumor
Testis	IgG4-related testicular disease
Skin and joints	
Skin	IgG4-related skin disease
Joints	IgG4-related synovitis

Abbreviation: IRC, IgG4-related cholangitis.

## History

**Fig. 1:**
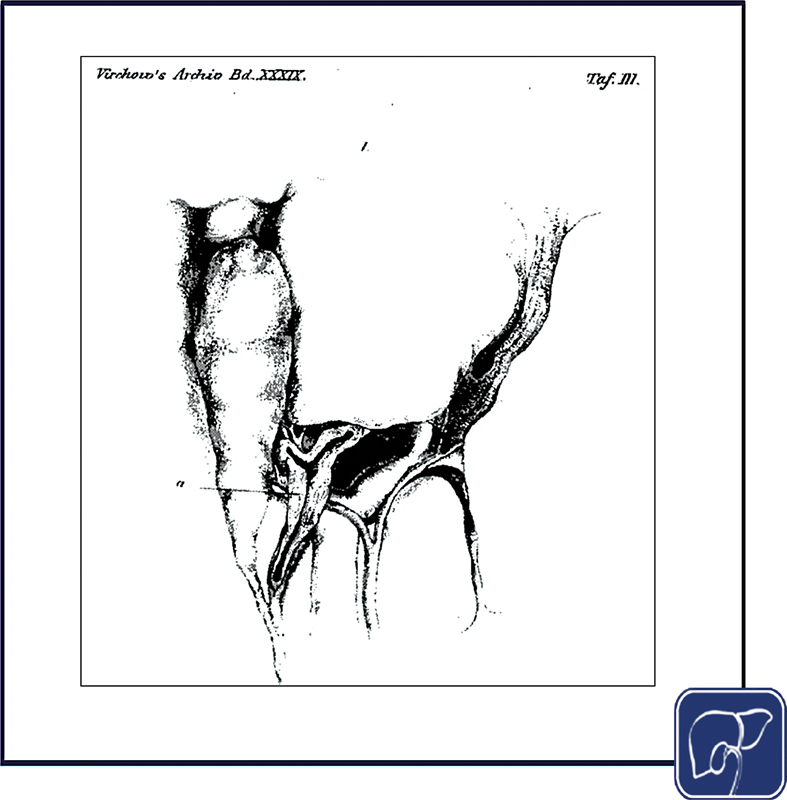
First published case of IgG4-related cholangitis? Early case description of a 60 years old factory employee with obstructive fibrosing cholangitis, long band-shaped bile duct wall thickening (3 mm) of the common bile duct relevant stenosis ( **A** ), and signs of chronic pancreatitis. 9

In 1866, a 60-year-old previously healthy and socially active factory employee from Basel (Switzerland) developed a severe cholangiopathy with deep jaundice and weight loss. 9 When he died before the end of the year, autopsy revealed an enlarged, dark brown–green discolored liver with a smooth surface, marked fibrotic longitudinal band-like bile duct wall thickening up to 3 mm of the common and the right and left hepatic ducts ( Fig. 1 ) without any microscopic evidence of malignancy, with cystic dilatation of the intrahepatic ducts without intrahepatic stenoses or pruning, a small gallbladder, an indurated and enlarged pancreas, but a barely enlarged spleen and no evidence of colitis. With today's knowledge, these well-documented findings appear most compatible with IRC and type 1 autoimmune pancreatitis (AIP) as the most frequent manifestations of IgG4-RD of the digestive tract in an elderly male exposed to industrial gases 10 rather than a first description of primary sclerosing cholangitis (PSC) as speculated for decades in the literature. In the late 19th century, additional organ manifestations of—what we think today—IgG4-RD such as Mikulicz's disease (1892), Küttner's tumor (1896), or Riedel's struma (1896) were described. The combined appearance of sclerosing cholangitis with Riedel's struma and retroperitoneal fibrosis was reported in 1963. 11 In the late 1990s the first five men with a “sclerosing pancreato-cholangitis” today fulfilling the diagnostic criteria of IRC and type 1 AIP were described as responding well to glucocorticosteroids. 12 13 IgG4-RD was first described as a multiorgan fibroinflammatory autoimmune disease in 2003. 14 IgG4-related sclerosing cholangitis with or without hepatic inflammatory pseudotumor was described in 2004 15 and defined in 2007 as IgG4-associated cholangitis, 16 before the name was adapted to that of other organ manifestations of IgG4-RD ( Table 1 ), that is, IRC, to better distinguish IRC name wise from serum IgG4-positive PSC, a different disease entity with considerable malignancy risk in the digestive tract. 17 18

## Clinical Presentation and Diagnosis

IRC typically affects males above 50 to 60 years of age (80–85%). 1 3 Obstructive jaundice, substantial weight loss and episodes of upper abdominal pain or discomfort are leading signs and symptoms 1 3 while cholestasis-associated pruritus is only reported by a minority (e.g., 13% in a Japanese cohort). 19 Endocrine pancreatic insufficiency (i.e., type 3c pancreatogenic diabetes mellitus) and exocrine pancreatic insufficiency are often detected upon screening (e.g., HbA1c in blood; elastase in faeces) due to the frequent association of IRC with type 1 AIP (∼90%). 3 20 21 Fever or night sweats are not regularly seen in adults with IRC and could alternatively be symptoms of bacterial superinfection in IRC or of an occult underlying malignancy. The clinical presentation of IRC may mimic other hepatobiliary diseases such as PSC and cholangiocarcinoma (CCA), but also very rare conditions like fibrohistiocytic pseudotumors, follicular cholangitis or sclerosing cholangitis with granulocytic epithelial lesion (SC-GEL). 1 No single validated diagnostic test is available to accurately diagnose IRC and exclude CCA and other mimics of IRC. Up to 30% of individuals with IRC, mainly with accompanying inflammatory pseudotumors, undergo unnecessary, often extended abdominal surgery for suspected malignancy (hemihepatectomy, pylorus-preserving pancreatoduodenectomy [PPPD]) before the diagnosis of IRC is made histopathologically. 3 21 22 23 Resection specimens in 10 to 15% of these surgical procedures may disclose fibroinflammatory lesions without malignancy mimicking malignant disease. In a considerable part of these patients, IgG4-RD can explain the preoperative findings leading to major surgery. 21 22 23 Hepatic inflammatory pseudotumors in the context of IRC have been described since 2004 and their histopathological similarity with IRC allowed the conclusion of a disease entity of IRC with/without inflammatory pseudotumors. 15 24 Glucocorticosteroid responsiveness was comparable to the inflammatory pseudotumors found in the pancreas in association with type 1 AIP. 15 25 Thus, hepatic inflammatory pseudotumors with histomorphological features of IRC are widely regarded as one feature of IRC.

**Fig. 2:**
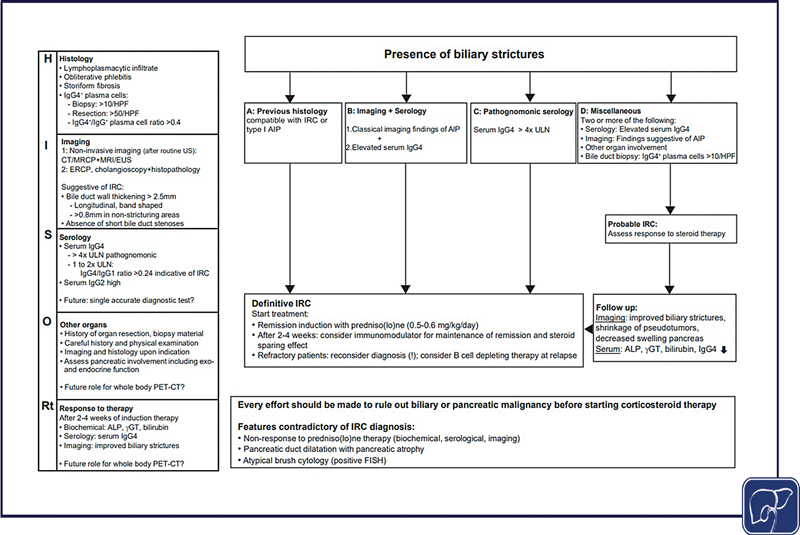
Diagnosis of IgG4-related cholangitis according to modified HISORt criteria. 1 Individuals with biliary strictures at cholangiography who are suspected of IRC should undergo analysis according to the HISORt criteria (left column). Depending on the results, the flow diagram on the right can be followed. The diagnosis of a “definitive IRC” can be assumed for individuals falling into category A, B, or C. For these, corticosteroid therapy is started and an immunomodulator is added when an early therapeutic response is documented and corticosteroids are tapered. Individuals falling into category D are defined as “probable IRC” and should be given a trial of corticosteroid therapy and have their response assessed. Every effort should be undertaken to rule out either biliary or pancreatic malignancy. 1 AIP, autoimmune pancreatitis; ALP, alkaline phosphatase; CT, Computed tomography; ERCP, endoscopic retrograde cholangiopancreaticography; EUS, endoscopic ultrasonography; γGT, gamma-glutamyl transferase; FISH, fluorescence in situ hybridization; HISORt, histology, imaging, serology, other organs, response to therapy; HPF, high-power field; IRC, IgG4-related cholangitis; MRCP, magnetic resonance cholangiopancreaticography. PET-CT, positron emission tomography-computed tomography; ULN, upper limit of normal.

The HISORt criteria are regarded as diagnostic standards and comprise histology (H), imaging (I), serology (S), other organ manifestations of IgG4-RD (O), and response to glucocorticosteroid therapy (Rt). 21 Fig. 2 provides a modified HISORt-based diagnostic work-up for IRC. 1 An overview of characteristics of the most relevant differential diagnoses of IRC, which are PSC, CCA, fibrohistiocytic pseudotumors, and follicular cholangitis, has recently been described. 1 To distinguish IRC from PSC, advanced age, professional history (blue-collar work?), absence of IBD, long band-shaped bile duct strictures rather than short strictures, single wall thickness >2.5 mm, and continuous bile duct wall thickening from the distal bile duct to the hilar region may help to diagnose IRC. To distinguish IRC from CCA when pathological specimens of adequate quality cannot exclude CCA, assessment of response to short-term glucocorticosteroid treatment for 2 to 4 weeks and biochemical and imaging control need to be considered ( Fig. 2 ).

**Fig. 3:**
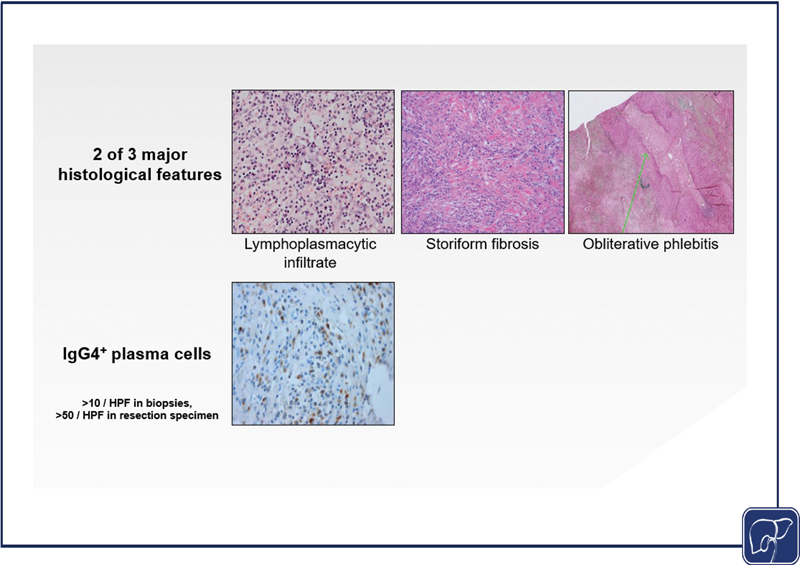
Histopathologic characteristics of IgG4-related cholangitis. Mandatory histopathological findings (two out of three) 5 of IRC in a liver resection specimen: Dense lymphoplasmacellular infiltrate (left image, H&E staining), storiform fibrosis (middle image, H&E staining), and obliterative phlebitis (right image, Elastica van Giesson staining). A dense infiltrate of >50 IgG4 ^+^ plasma cells per HPF demonstrated by immunohistochemistry (IgG4 ^+^ plasma cells colored brown after staining with an IgG4-specific monoclonal antibody; courtesy of Prof. Joanne Verheij). H&E, hematoxylin and eosin; HPF, high-power field.

Histology (H): In IRC, bile duct specimens show characteristic fibro-inflammatory lesions in the bile duct wall consisting of (i) a dense lymphoplasmacellular infiltration rich in IgG4 ^+^ plasma cells, CD4 ^+^ T lymphocytes and eosinophilic granulocytes, (ii) typical histopathological features such as obliterative phlebitis, and (iii) a cartwheel-shaped storiform fibrosis particularly in advanced stages ( Fig. 3 ). 5 26 The number of IgG4 ^+^ plasma cells and the ratio of IgG4 ^+^ /IgG ^+^ plasma cells per high power field (HPF) are less specific and may overlap with those in PSC or CCA. 5 For IRC, >10 IgG4 ^+^ plasma cells per HPF in biopsy specimens and >50 IgG4 ^+^ plasma cells per HPF in resection specimens as well as an IgG4 ^+^ /IgG ^+^ ratio >0.4 would fit with the diagnosis ( Fig. 3 ), 5 although ratios of >0.7 are more commonly seen in IRC. 27 The HPF with the highest number of cells in the specimen is decisive as IgG4 ^+^ cell distribution may be patchy. 5 As opposed to the volume of resection specimens available after major surgery for histopathological analysis, the low amount of available liver tissue after liver needle biopsy and the lack of depth of bile duct biopsies carry the risk of sampling errors and are technical shortcomings which may explain in part the inadequate sensitivity of liver and bile duct biopsies to assess at least the criterion of obliterative phlebitis. Papillary biopsies are controversial (e.g., risk of postbiopsy pancreatitis). 17 For the exclusion of CCA, adequate quality of pathological specimens of lesions is crucial.

**Fig. 4:**
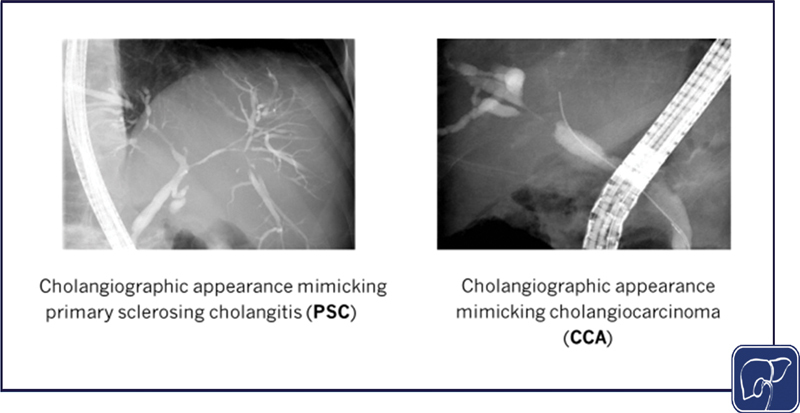
Findings at endoscopic retrograde cholangiography in IgG4-related cholangitis mimicking primary sclerosing cholangitis and cholangiocarcinoma.

Imaging (I): Magnetic resonance imaging (MRI)/magnetic resonance cholangiopancreaticography (MRCP), computed tomography (CT), endoscopic ultrasound (EUS), intraductal ultrasound (IDUS), or cholangioscopy may show bile duct strictures with wall thickening of the extrahepatic, perihilar, and/or intrahepatic bile ducts during imaging of the liver and biliary tree, and/or lesions suspicious for malignancy like inflammatory pseudotumors. 28 29 A recent multicentre analysis from Japan and the United States disclosed that—next to elevated serum IgG4—EUS and IDUS are useful imaging modalities for the diagnosis of IRC. The pattern of bile duct involvement has led to the differentiation of IRC into type 1 (distal stricture of the common bile duct only; half of the patients), type 2 (intrahepatic segmental or diffuse bile duct alterations and distal stricture of the common bile duct, with prestenotic dilatation; type 2a, or without prestenotic dilatation; type 2b), type 3 (hilar and distal stricture of the common bile duct), and type 4 (hilar stricture of the common bile duct). 30 Bile duct wall thickening with more band-shaped constrictions, 29 ( Fig. 1 9 ), or a single-wall common bile duct thickness >2.5 mm on MRI, 29 the absence of short bile duct strictures, 31 as well as circular symmetric wall thickness with smooth inner and outer margins of the bile duct wall and a wall thickness of >0.8 mm in nonstrictured areas on intraductal sonography 32 were all suggestive of IRC when compared with PSC or CCA, respectively. 1 Imaging may also disclose extra biliary abdominal organ involvement, particularly of the pancreas as a characteristic feature of IRC. The pancreas might appear enlarged and sausage-shaped, hypoechoic on ultrasound, with an edematous swelling of the surrounding fat tissue (halo) and multifocal strictures of the pancreatic duct. Inflammatory pseudotumors suspicious for pancreatic malignancy, gallbladder wall thickening, and renal abnormalities may also be disclosed. The additional usefulness of positron emission tomography with CT (PET-CT) for diagnosis and evaluation of treatment response in IgG4-RD remains unclear at this moment (also considering costs and radiation exposure). 17 Starting diagnostics after routine abdominal ultrasound in cholestasis 33 with noninvasive imaging modalities such as contrast-enhanced MRI/MRCP or CT is advised. 33 More “invasive” imaging methods such as EUS and ERCP ( Fig. 4 ) with brush, biopsy, or cholangioscopy (if needed) would follow to obtain pathological samples from sites suspected of malignancy.

Serum biomarkers (S): Elevated serum IgG4 is described in approximately 75 to 80% of individuals with IRC. 1 2 3 34 However, only an elevation of more than four times the upper limit of normal (ULN) is regarded as pathognomonic, 35 36 as moderately elevated (mostly 1–2× ULN) IgG4 serum levels are also observed in PSC (∼15%), CCA, chronic pancreatitis of different origin or pancreatic adenocarcinoma. 3 When serum IgG4 levels are >1.4 g/L (ULN) and <2.8 g/L (2× ULN) in sclerosing cholangitis, incorporating the IgG4/IgG1 ratio with a cut-off of 0.24 improves the positive predictive value and specificity to distinguish IRC from PSC. 36 Typical biochemical profiles of individuals with IRC before the start of medical treatment are exemplified by a cohort of 29 consecutive Dutch individuals with IRC fulfilling the HISORt criteria for IRC ( Table 2 ). 37 The majority showed a cholestatic profile with prominent alkaline phosphatase and glutamyltransferase, but only mild elevation of ALT and AST (except for acute biliary obstruction when ALT and AST can be markedly elevated). Improvement of cholestatic serum markers such as alkaline phosphatase and conjugated bilirubin are used to determine treatment response next to imaging for hepatobiliary alterations but have not been adequately validated so far. Carbohydrate antigen 19–9 (CA19–9) does not allow differentiation of IRC from CCA or pancreatic adenocarcinoma since CA19–9 serum levels may be markedly elevated in all conditions. 38 Still, the treatment response of CA19–9 to glucocorticosteroids may markedly differ between IRC and CCA (personal observation) and should be further studied prospectively. Newer blood (not serum) biomarkers are of potential diagnostic value, although diagnostic accuracy and feasibility in routine clinical practice remain to be validated. We identified affinity maturated, class-switched IgG4 ^+^ B cell receptor clones by next-generation sequencing in blood and affected tissue of people with IRC including multiorgan IgG4-RD. 39 The detection of these clones probably allows for reliable differentiation of active IRC from PSC, CCA, and pancreatic adenocarcinoma. 39 Our data were then supported by observations of circulating plasmablast counts in individuals with multi-organ involvement of IgG4-RD mainly outside the digestive tract. 40 41 In contrast to the above-summarized studies, a diagnostic value of serum IgG4/IgG RNA ratio for IRC versus pancreatobiliary malignancies could not be shown. 42 A recent nuclear magnetic resonance-based metabolomic approach to distinguish IRC from PSC serum showed remarkable accuracy, but may need extramural validation; its potential to distinguish IRC and PSC from pancreatobiliary malignancies could also be studied. 43

**Table 2:** Biochemical markers of 29 consecutive Dutch individuals with IRC before the start of medical treatment (in several cases after major surgery leading to the diagnosis of IRC) 37

Test	ULN	Mean	SD	Min	Max
Bilirubin (mmol/L)	17	31	44	4	201
AST (U/L)	40	69	65	18	347
ALT (U/L)	45	93	91	19	406
ALP (U/L)	120	405	292	68	1,185
GT (U/L)	60	499	457	9	1,833
CA 19–9 (U/L)	37	148	269	10	928
IgG4 (g/L)	1.4	6.7	6.7	0.9	24.8

Abbreviations: IRC, IgG4-related cholangitis; SD, standard deviation; ULN, upper limit of normal.

Other organ involvement (O): The strong association of IRC with type 1 AIP (∼90%) and type 1 AIP with IRC (30–60%) is mentioned above. Table 1 summarizes also other organs and sites of manifestations of IgG4-RD. A carefully taken medical history and meticulous physical examination may disclose former and present extrahepatic manifestations of IgG4-RD such as IgG4-related sialadenitis or prostatitis which may have gone undiagnosed. Histopathological revision for IgG4-RD of formerly taken biopsies from extrahepatic organs should be considered. The prominent involvement of glands and alkaline fluid-producing tissues like the pancreas, bile ducts, salivary and lacrimal glands, prostate, testes or vagina led us to speculate on IgG4-RD as a secretory disorder such as primary biliary cholangitis (PBC), 44 a progressive cholestatic immune-mediated liver disease of the smallest bile ductules which shows a comparable extrahepatic organ involvement with impairment of potentially protective bicarbonate secretion not only of the intrahepatic biliary tree, 45 46 47 48 but also the salivary and lacrimal glands (“dry eye and dry mouth”), thyroid, pancreas, vagina (vaginal dryness) or testes. 4 44 49

Response to therapy (Rt): Glucocorticosteroids are recommended as first-line treatment of IRC, given at 30 to 40 mg/day (0.5–0.6 mg/kg) predniso(lo)ne daily 3 17 33 with a nearly 100% response rate for clinical, biochemical (bilirubin, alkaline phosphatase, and γGT, elevated CA19–9), and/or imaging findings within 2 to 4 weeks of predniso(lo)ne therapy. Response to glucocorticosteroid therapy is regarded as a diagnostic hallmark in the distinction of IRC from CCA and PSC. The biological half-life of serum IgG4 is approximately 21 days, thus a moderate decrease might also be observed during this initial treatment period. An IgG4-RD responder index (RI) has previously been introduced by rheumatologists for research purposes to quantify response in systemic IgG4-RD. 50 51 Whether the IgG4-RD RI has additive value in the analysis of therapeutic response in IRC and type 1 AIP as major IgG4-RD manifestations of the digestive tract is unclear as only one case of the original IgG4-RD cohort 50 and one biliary and one pancreatic case of the IgG4-RD validation cohort 51 were included for validation of the IgG4-RD RI.

In conclusion, we consider the modified HISORt criteria ( Fig. 2 ) as the actual standard for the diagnosis of IRC in clinical practice. 1 21 The diagnosis of a “definite IRC” is accepted and glucocorticosteroid therapy can be started when in addition to cholangiographic features of sclerosing cholangitis (A) IgG4-RD of either the bile ducts or pancreas is histologically proven (e.g., after hemihepatectomy or PPPD) 5 ; or (B) imaging findings typical for AIP (sausage-like shape, focal pancreatic mass/enlargement without pancreatic duct dilatation, multiple pancreatic masses, focal pancreatic duct stricture without upstream dilatation, and pancreatic atrophy) are supported by elevated serum IgG4 levels 21 ; or (C) IgG4 serum levels are >4× ULN. 1 35 36 A fourth group (D) categorized as having “probable IRC” would need to fulfill two or more of the following criteria: elevated serum IgG4, imaging findings suggestive of type 1 AIP, other organ involvement, or a bile duct biopsy showing >10 IgG4 ^+^ plasma cells per HPF. A time-limited course of glucocorticosteroids over, for example, 2 to 4 weeks is justified here and should only be continued when treatment response is documented ( Fig. 2 ). Nonresponse to glucocorticosteroids seriously questions a diagnosis of IRC. Still, long-lasting fibrotic bile duct strictures may need endoscopic intervention with balloon dilatation or short-term stenting and histological exclusion of CCA.

There clearly is an unmet need for validated diagnostic tests (in addition to the helpful and pragmatic HISORt criteria) that are able to accurately diagnose IRC and distinguish it from PSC, pancreatobiliary malignancies, and other rare cholangiopathies including fibrohistiocytic pseudotumors, follicular cholangitis, or SC-GEL ( Table 2 ).

## Pathogenesis of IgG4-Related Cholangitis (and IgG4-RD)

Genetic factors: Like in other immune-mediated diseases including fibrosing cholangiopathies such as PBC or PSC, several HLA and non-HLA gene variants have been identified as risk genes for various manifestations of IgG4-RD, but not specifically IRC in association studies as summarized by Ishikawa and Terao. 52 Among these, HLA-DRB1 was identified as a risk gene in a Japanese genome-wide association study 53 suggesting that antigen presentation and recognition may play a critical role in the pathogenesis of IgG4-RD. Among the non-HLA genes identified, 53 the FCGR2B gene product FCγR2B is of interest as it represents the only FCγ receptor family member expressed in B cells and is thought to contribute to the elimination of autoreactive B cells; thus, FCGR2B gene variants might increase susceptibility to autoimmunity. 54 IL1R1 variants were recently associated with IgG4-related periaortitis/periarteritis. 55 Variants of CTLA4 coding for an inhibitory receptor expressed on activated memory T cells were associated with type I AIP in a small IgG4-RD cohort. 56 Several other mostly small-scaled association studies limited to Asian individuals have identified additional risk loci mostly for specific organ manifestations of IgG4-RD and need further validation. 52 For IRC cohorts, comparable findings are not yet reported.

Environmental and occupational toxins: We identified “blue-collar work” with long-term, often lifelong exposure to industrial contaminants as a potential risk factor in independent cohorts from the Netherlands and the UK. 57 Our subsequent extended prospective analysis disclosed vapors, dust, industrial gases, fumes (VDGF), and asbestos as independent risk factors for the development of IRC and type I AIP when compared with sex- and age-matched controls (odds ratio [OR]: 3.66; confidence interval: 2.18–6.13; *n* = 404; *p* < 0.0001). 58 Typical work environments included exposure to industrial vapors/dusts/gases/fumes in the oil and metal industry, automobile repair, truck driving, painting, or woodworking. These strongly male-dominated work profiles led us to speculate that they may contribute to the remarkable overrepresentation of men (80–85%) among people with IRC and type 1 AIP 57 58 as opposed to various other organ manifestations. Notably, asbestos was not only identified in our quite large cohort as an independent risk factor for IRC and type 1 AIP, 58 but also as a risk factor for largely unclassified retroperitoneal fibrosis in the past in case series 59 supporting our recent finding. Remarkably, also cigarette smoking was recently identified to be more common among a large group of patients with different organ manifestations of IgG4-RD mainly outside the digestive tract, particularly in patients with IgG4-related retroperitoneal fibrosis. 60 Protection against VDGF and asbestos exposure and smoking appear as valuable preventive measures to lower the risk of IgG4-RD and potentially its severity next to the risk of malignancies of different organs induced by these toxins. A most recent report from Mexico identified exposure to biomass fuel as a risk factor mainly for IgG4-RD of the head, neck, and thorax 61 in line with our observation of woodworkers being at risk for IgG4-RD. 58 As biomass fuel is commonly used in developing countries for heating, the balanced female/male ratio in the Mexican cohort would support exposure to specific environmental toxins as the relevant initiator of IgG4-RD 61 comparable to the strongly male-dominated blue-collar workers of our IRC/AIP cohorts. How could occupational toxins be involved in the pathogenesis of IgG4-RD? They might directly damage tissues causing exposure of autoantigens and damage-associated molecular patterns (DAMPs) to the immune system thereby initiating an autoimmune response in genetically predisposed individuals. They could also trigger autoreactive B and T cells via molecular mimicry or cause genetic and epigenetic changes stimulating autoimmunity. Alternatively, they could also induce the development of malignancies as precursors of IgG4-RD. 1 62

Microbiota: Faecal analysis recently revealed reduced α diversity, shifted microbial community, and distinct host-microbe associations in IRC and PSC when compared with healthy controls. 63 These and other analyses do not allow at present to adequately judge a pathogenic role of microbiota in the pathogenesis of IRC.

Malignancies: Enhanced risk of malignancy in IRC/AIP has been reported 20 and malignancies prior to the onset and diagnosis of IRC have been discussed as potential (co-)initiators of IRC. 1 In a recent retrospective Japanese survey comprising 924 individuals with IRC, 139 developed prior to, simultaneous with, or after the diagnosis of IRC 149 malignancies mainly of the gastrointestinal, urinary, or respiratory tract (69%) of which 48% had been detected before or within 3 months of the diagnosis of IRC. 64 Cancer-induced (or chemo-/radiotherapy-related tumor destruction-induced) autoimmunity is discussed as one potential initiator of immune-mediated inflammatory diseases 65 and might explain an abnormal immune response against tumor autoantigens in antigen-expressing organs also in a subgroup of genetically predisposed individuals suffering from IRC and IgG4-RD. Alternatively, considering that occupational toxins such as VDGF (e.g., diesel exhaust 66 67 ) or asbest are in part known as risk factors not only of IRC and IgG4-RD, but also of various malignancies, 58 a common culprit might trigger autoimmunity and malignancy in subgroups of individuals with IRC and IgG4-RD.

Autoantigens: IgG4 is the least abundant subclass of IgG in human serum. 68 IgG4 responses have a blocking effect, either on the immune response induced by other IgGs or on the target protein of IgG4 which can be beneficial or detrimental. 68 When we found dominant oligoclonal IgG4 ^+^ B cell populations in sera and affected tissue of patients with IRC/AIP, this raised our suspicion that the immune response in IgG4-RD could be targeting specific autoantigens. 39 Our findings were then supported by the finding of oligoclonal IgG4 ^+^ plasmablasts in the blood of individuals with IgG4-RD of various organ manifestations mainly outside the digestive tract. 40 Meanwhile, various autoantigens and autoantibodies have been described in IgG4-RD. 1 69 Most of these autoantibodies are not disease-specific. 1 69 In IRC, the presence of IgG4-specific autoantibodies against annexin A11, 37 49 laminin 511-E8, 70 71 galectin-3 72 73 and prohibitin 1 73 has been confirmed with expression of autoantigens in cholangiocytes. 1 The potential pathogenic relevance of these autoantibodies has been strongly supported by the seminal observation that mice injected with IgG isolated from sera of individuals with IgG4-RD develop typical organ lesions. 74 Furthermore, patients who are positive for several of the above-mentioned autoantibodies were shown to have a more severe disease. 75 The pathogenicity of IgG subtypes markedly differs, 68 and data rather suggest a damaging role for IgG1 by eliciting an excessive immune response after binding of the autoantibody and/or blocking the function of the targeted autoantigen and a potentially protective role for IgG4 autoantibodies against damaging IgG1 effects. 37 74 Still, also IgG4 autoantibodies could potentially contribute to the pathogenesis by directly blocking the function of the targeted autoantigen. 68

**Fig. 5:**
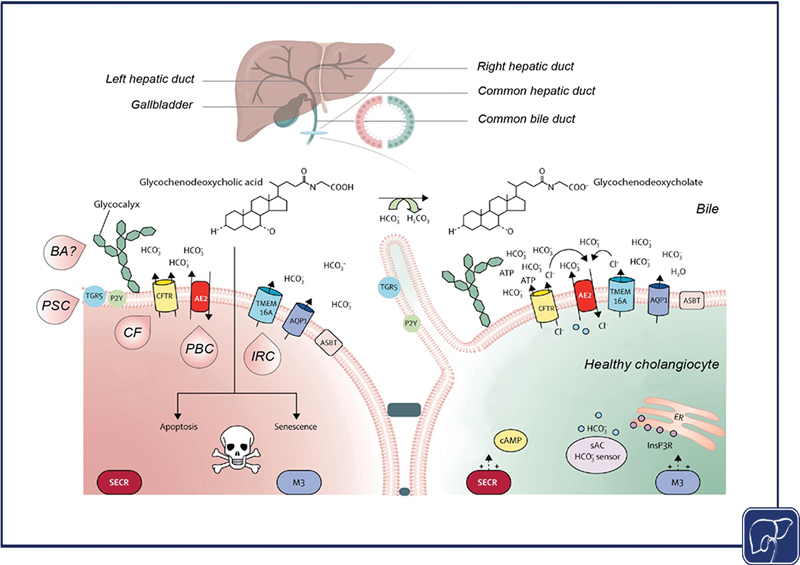
The “biliary bicarbonate umbrella” hypothesis (image adapted from Dyson et al 18 and Beuers et al 46 ). On the right a healthy cholangiocyte expressing adequate cellular machinery to secrete bicarbonate and deprotonate the hydrophobic bile acid GCDC to its corresponding bile salt. On the left, a damaged cholangiocyte potentially caused by GCDC diffusion across the plasma membrane due to dysfunction of the “biliary bicarbonate umbrella” as described in the immune-mediated and genetic cholestatic liver diseases PBC, PSC, IRC, CF, and BA, respectively. 48 49 71 82 120 AE2: anion exchanger 2; ASBT: apical sodium dependent bile salt transporter; ATP: adenosine trisphosphate; AQP1: aquaporine 1; BA: biliary atresia; Ca ^2+^ : calcium; cAMP: cyclic adenosine monophosphate; CF: cystic fibrosis; CFTR: cystic fibrosis transmembrane conductance regulator; Cl ^−^ : chloride; ER: endoplasmatic reticulum; HCO _3_^−^ : bicarbonate; H _2_ CO _3_ : carbonic acid; InsP _3_ R _3_ : type III inositol 1,4,5-trisphosphate receptor; M3: muscarinic receptor 3; PBC: primary biliary cholangitis; PSC: primary sclerosing cholangitis; sAC: soluble adenylyl cyclase; SECR: secretin receptor; TGR5: Takeda G-protein coupled receptor 5; P2Y12: purinergic G protein-coupled receptor type 12; TMEM16A: anoctamin-1 (ANO1).

The first identified IgG4/IgG1 target autoantigen in IRC was annexin A11. 37 Annexin A11 mediates, among other functions, Ca ^2+^ -dependent membrane trafficking in different tissues including the major organ manifestations of the digestive tract in IgG4-RD: the biliary tree 37 49 and pancreas. 76 In the biliary tree, this process seems important for the maintenance of an apical defense mechanism against the toxic effects of biliary glycine-conjugated bile acids in humans, referred to as the “biliary HCO _3_^−^ umbrella.” 46 47 77 78 79 Glycine-conjugated bile acid permeation due to an impaired biliary HCO _3_^−^ umbrella likely contributes to the progressive bile duct destruction found in immune-mediated cholangiopathies such as PBC, 45 48 80 81 PSC, 82 or IRC 1 49 ( Fig. 5 ). Annexin A11 is predominantly expressed in cholangiocytes within the human liver, 49 the cell type that is mainly affected in IRC. Furthermore, in human cholangiocytes annexin A11 mediates the apical membrane insertion of the Ca ^2+^ -sensitive Cl ^−^ channel anoctamin-1 (ANO1; also known as TMEM16A). ANO1 and the apical cAMP-sensitive cystic fibrosis transmembrane conductance regulator (CFTR) are crucial for the formation of a stable biliary HCO _3_^−^ umbrella as they create the Cl ^−^ gradient necessary for apical HCO _3_^−^ secretion via the major human Cl ^−^ /HCO _3_^−^ exchanger, anion exchanger 2 (AE2). Membrane transfer and insertion of ANO1 by annexin A11 was markedly impaired in human cholangiocytes after incubation with cholestatic IRC patient serum with high titers of anti-annexin A11 IgG1 and IgG4 autoantibodies, but not after incubation with cholestatic PSC control sera. 49 Thus, an IgG1/IgG4-mediated autoreactivity against annexin A11 might contribute to the pathogenesis of IRC by weakening the biliary HCO _3_^−^ umbrella 49 ( Fig. 5 ).

Autoantibodies against laminin 511-E8 were first identified in individuals with type I AIP 70 and confirmed by us also in a subgroup of individuals with IRC. 71 Laminin 511-E8 is a heterotrimeric extracellular matrix protein that promotes cholangiocyte differentiation of human induced pluripotent stem cells and, thereby, upregulation of secretory components of the “biliary bicarbonate umbrella,” such as the apical cAMP-sensitive Cl ^−^ /HCO _3_^−^ channel CFTR, the G protein-coupled bile acid receptor 1 (GPBAR1, also known as TGR5) and the basolateral secretin receptor with the effect of increased fluid secretion into cholangiocyte cysts. 83 Laminin 511 also stabilizes endothelial barrier properties and blocks leukocyte extravasation. 84 Given the apparent cholangiocyte barrier dysfunction in IRC, we have recently identified a protective role of laminin 511-E8 in human cholangiocytes in vitro against T lymphocyte-induced barrier dysfunction, toxic bile acid permeation, and cholangiocyte apoptosis. 71 These findings made it tempting for us to speculate that a decreased epithelial barrier function with attraction of immune cells and impaired bicarbonate secretion as a result of dysfunction of laminin 511 by autoantibody binding could potentially be a common systemic pathogenic mechanism in a subset of patients with IgG4-RD. 71

We also confirmed the presence of autoantibodies against the carbohydrate-binding lectin galectin-3 and the scaffold proteins prohibitin 1 and 2 in the blood of a subset of individuals with IRC 73 described before for other organ manifestations of IgG4-RD. 72 Gene-specific knockdown, pharmacological inhibition, and recombinant protein substitution did not clearly disclose a protective role of these autoantigens in human cholangiocytes. 73 The involvement of these autoantibodies in processes surpassing cholangiocellular epithelial secretion remains to be elucidated.

Autoantibodies against carboanhydrase 2 (CA2) and 4 (CA4) have early been described in AIP and IgG4-RD. 1 69 In human cholangiocytes, expression of CA2, CA5b, CA9 and CA12 is identified. 85 Subsets of IRC and PSC patients have anti-CA2 and anti-CA5b autoantibodies that functionally inhibit human recombinant CA isoforms, thereby potentially rendering human cholangiocytes more susceptible to bile acid-induced damage. 85 Additional data are upcoming to further substantiate these potentially relevant findings.

The innate immune system: Activation of the innate immune system is a prerequisite for the formation of autoantibodies by the adaptive immune system, one of the hallmarks of IRC and IgG4-RD. 69 86 The crosstalk between innate and adaptive immune systems is extensive in IgG4-RD and antigen presentation by the innate immune system has been hypothesized to initiate the aberrant B and T cell responses in IgG4-RD. 87 The predilection of IgG4-RD to affect secretory epithelia and glands of the digestive tract such as the pancreas, bile ducts, or salivary glands that are exposed to endogenous and environmental stressors is still enigmatic. It has been speculated that damage-associated molecular patterns and pathogen-associated molecular patterns (PAMPs; or microbe-associated molecular patterns [MAMPs]) could activate the innate immune system in IgG4-RD. 86 This might be due to long-term exposure to environmental or occupational toxins, bacteria or other microbes and self-antigens that could function as DAMPs/MAMPs/PAMPs, possibly through mechanisms of molecular mimicry, for example, among microbes and target antigens. When in an early experimental study female IL10 ^−/−^ mice and MRL/Mp mice were injected intraperitoneally twice a week with a known activator of the innate immune system (polyinosinic polycytidylic acid) they developed lesions typical of type I AIP and IRC, but only MRL/Mp mice developed also sialadenitis with IgG4-typical histopathological features, in conjunction with the formation of autoantibodies directed against lactoferrin, carbonic anhydrase 2 (CA2) and pancreatic secretory trypsin inhibitor. 88 This study suggested that IRC/AIP characteristics are inducible by activation of the innate immune system and the genetic background may determine susceptibility to extrabiliopancreatic involvement. 88 Recent findings indicate that the innate immune system is activated via toll-like receptors and nucleotide-binding oligomerization domain-like receptors on monocytes, CD-163+ M2 macrophages, and basophils in various organ manifestations of IgG4-RD 86 89 leading to increased production of IgG4 by plasmablasts via B cell activating factor (BAFF), IL-33 and IL-13. 90 Candidates for the activation of the innate immune system in IRC and IgG4-RD are discussed above.

The adaptive immune system—T cells: Involvement of regulatory T cells (Tregs), follicular T helper 2 (Tfh2), peripheral T helper (Tph), CD8 ^+^ and CD4 ^+^ signaling lymphocytic activation molecule (SLAMF7 ^+^ ) cytotoxic T cells (CTLs) in IgG4-RD has recently been summarized. 1 91 Tregs play an important role in the promotion of IgG4 class switching and fibrosis. They regulate self-tolerance and secrete the anti-inflammatory cytokines IL-10 and TGF-β. In IRC, Tregs show increased infiltration in the bile ducts correlating with the amount of IgG4-positive B cells. 92 Tfh2 cells stimulate antigen-specific B cell proliferation toward IgG4 secreting plasmablasts, somatic hypermutation, isotype class switching, germinal center development, and secretion of the cytokines IL-4 inducing isotype class switching and IL-21 allowing plasmablast and plasma cell differentiation. 93 The Tfh2 cell subset is increased in blood and positively correlates with disease activity, number of affected organs, and serum IgG4 levels and decreases after glucocorticosteroid treatment, 94 also in IRC/AIP. 95 T peripheral helper (Tph) cells may play a more critical role than Tfh2 cells in IRC. They form ectopic lymphoid structures outside lymph nodes that could maintain the inflammatory process and release cytotoxic agents such as granzyme and perforin leading to tissue damage. Their numbers in blood are increased, correlate with serum IgG4 levels and disease severity, and are glucocorticosteroid-responsive. 96

Two types of cytotoxic T lymphocytes (CTLs) have been identified and held responsible for cellular damage in IgG4-RD, although not yet confirmed for IRC: (1) oligoclonal expanded CD4 ^+^ SLAMF7 ^+^ CTLs have been shown in blood and affected tissue. 97 98 They are able to secrete granzyme A, perforin, and IFN-γ to kill target cells and secrete cytokines such as IL-1β. 97 98 Notably, they do not express CD20 but still are reduced upon the anti-CD20 antibody rituximab suggesting that rituximab-sensitive B cells can regulate effector/memory CD4 ^+^ T cells in IgG4-RD. 97 (2) The presence of dominant oligoclonal subsets of effector/memory CD8 ^+^ T cells with an activated and cytotoxic phenotype in blood and infiltration of granzyme A-expressing CD8 ^+^ CTLs in disease tissues was recently demonstrated. 99 These CD8 ^+^ CTLs preferentially induce apoptosis in mesenchymal rather than endothelial or immune cells. 99

The adaptive immune system—B cells: The B cell lineage including plasmablasts, plasma cells, and memory B cells has a central role in IgG4-RD. 87 But it remains unclear whether the role of IgG4 ^+^ B cells is protective, pathogenic, or both for the attacked organ in IgG4-RD. 69 87 91 In IRC- and type 1 AIP-dominated IgG4-RD, we have shown that the B cell receptor repertoire of patients contains oligoclonal expansions of IgG4 ^+^ B cells/plasmablasts which exhibit signs of affinity maturation, suggesting an antigen-driven response. 39 Our studies and those of Mattoo et al showed in independent cohorts with diverse organ manifestations of IgG4-RD that these IgG4 ^+^ plasmablasts disappear upon treatment of IgG4-RD. 39 40 Notably, the IgG4 ^+^ plasmablasts that reappeared at relapse were distinct from the ones present during the initial peak of disease activity. 40 This indicated to Mattoo et al that new naïve B cells were recruited by CD4 ^+^ T cells to undergo repeated rounds of mutation and selection driven by a self-reactive disease process.

IgG4 ^+^ B cells could produce potentially pathogenic IgG4 autoantibodies in IRC/AIP and IgG4-RD. 37 49 70 71 In addition, IgG4 ^+^ B cells could stimulate and reactivate CD4 ^+^ CTLs. The latter would be supported by the observation that rituximab treatment reduces CD3 ^+^ T cells. 100 They also could actively affect tissue fibrosis 101 corresponding with the finding that rituximab treatment decreased enhanced liver fibrosis scores and myofibroblast volume in people with IgG4-RD. 100 On the other hand, IgG4 produced by IgG4 ^+^ B cells could function in IRC, type 1 AIP, and IgG4-RD with other organ manifestations to dampen an excessive IgG1-mediated immune response. 37 49 71 74 Finally, circulating CD21 ^low^ memory B cells have been shown to be increased in patients with IgG4-RD. 102 Notably, a rise in circulating memory B cells was the only biomarker of all the elements of the B cell lineage studied to accurately predict disease relapse after glucocorticosteroid treatment. 103

## Therapy of IRC

The natural course of IRC without therapeutic intervention can lead to obstructive jaundice, bacterial cholangitis, liver abscesses, cholecystitis, pruritus, biliary fibrosis, cirrhosis, and death. Thus, adequate treatment is mandatory. Treatment aims are induction and long-term maintenance of remission. 1 2 3 33 104

Induction of remission: Predniso(lo)ne (0.5–0.6 mg/kg/day) is recommended as the first-line therapy for untreated active IRC. Treatment response should be evaluated after (2 to) 4 weeks, prior to predniso(lo)ne tapering, by clinical, biochemical, and/or radiological criteria (LoE 5, strong recommendation, 100% consensus). 33 Predniso(lo)ne is progressively tapered down with 5 mg every 2 weeks until a maintenance dose of ≤7.5 mg/day is reached. 33 Medium-dose prednisone (0.5–0.6 mg/kg) has been shown to be as effective as high-dose prednisone (0.8–1 mg/kg/day) for inducing remission in IgG4-RD. 105 Still, the number and kind of organs involved and the severity of organ damage on one hand and the tolerability of glucocorticosteroids (e.g., diabetes, osteoporosis) in mainly elderly affected individuals may urge for modifications of the maximum dose and the rate of tapering down. 33 104 A response to glucocorticosteroid therapy is nearly 100% when the diagnosis of IRC and other manifestations of IgG4-RD is correct ( Fig. 2 ). Yet, disease recurrence after tapering and cessation of treatment (after 3–6 months) is seen in at least 50% of affected individuals. 1 2 21 The major anti-inflammatory and antifibrotic mechanisms of action of glucocorticosteroids responsible for the marked clinical, biochemical, radiological, and histological improvement in IRC and IgG4-RD are not yet clearly unraveled on the molecular level. A notable finding potentially adding to the impressive glucocorticosteroid effects in IgG4-RD is the overexpression of glucocorticosteroid receptor in various organs affected by IgG4-RD including salivary glands and kidneys, but has not yet been studied in the hepatobiliary tract for IRC. 106

As discussed above, B cells appear to play a central role in IgG4-RD. Notably, oligoclonal expansion of IgG4 ^+^ B cells became undetectable after 4 and 8 weeks of prednisolone treatment in IRC, 39 and elevated circulating plasmablasts and plasma cells were depleted by glucocorticosteroids in IgG4-RD in parallel with clinical improvement 107 whereas memory B cells increased, but CD19 ^+^ and CD20 ^+^ B cells were not affected. 107 This might in part explain why relapse of IgG4-RD after the stop of glucocorticosteroid treatment in at least 50% of treated individuals may be successfully treated with anti-CD19 (e.g., ibalizumab) and anti-CD20 (e.g., rituximab) strategies to adequately reduce precursor CD19 ^+^ and CD20 ^+^ B cells.

Maintenance of remission: Maintenance treatment of IRC and IRC/AIP is suggested with glucocorticosteroid-sparing immunosuppressants for up to 3 years and potentially beyond (e.g., azathioprine, 6-mercaptopurine, mycophenolate mofetil) starting during predniso(lo)ne tapering, to reduce the risk of IRC relapse. Rituximab can alternatively be considered when relapse has occurred (LoE 5, weak recommendation, 100% consensus). 33 After securing the initial glucocorticosteroid response to confirm the diagnosis of IgG4-RD, immunomodulators have been added to glucocorticosteroids in various treatment protocols for IgG4-RD similar to treatment protocols for autoimmune hepatitis 108 109 to lower the cumulative predniso(lo)ne dose and reduce the risk of relapse. 1 One randomized trial for AIP, observational studies with azathioprine, iguratimod, and methotrexate and clinical trials with mycophenolate mofetil, leflunomide and cyclophosphamide for IgG4-RD support these protocols. 1 At present, there is no documented evidence to advise on one immunomodulator above the others. In addition, a recently performed network analysis described that glucocorticosteroids and immunomodulators reached higher remission rates in IgG4-RD than glucocorticosteroids only (OR: 3.4), immunomodulators only (OR: 55.3) or rituximab induction treatment only (OR: 7.4). 110 Still, rituximab maintenance treatment had the lowest relapse rate (OR: 0.10). 110

The anti-CD20 antibody rituximab has become a preferred treatment for long-term remission of diverse organ manifestations of IgG4-RD by rheumatologists, internists, and other specialists in high-budget healthcare systems caring for patients with IgG4-RD. Also for IRC, rituximab has been reported to be efficient in long-term remission in a single-arm observational study and a retrospective case analysis. 111 112 However, a potentially increased and prolonged risk of infections after B cell depletion must be considered 112 which might be critical for IRC (different from other organ manifestations) with infectious IRC complications of bacterial cholangitis, cholecystitis, or liver abscesses.

Novel therapeutic approaches: In addition to rituximab, various new medications, mainly monoclonal antibodies, were developed to more specifically dampen IgG4-related autoimmune reactions and autoantibody effects. 1 Dr. Stone and colleagues from American, Asian, and European Centers were meanwhile able to document in the remarkable phase 3, multicenter, double-blind, randomized, placebo-controlled MITIGATE trial in 135 patients with IgG4-RD that the anti-CD19 monoclonal antibody Inebilizumab reduced the risk of relapse of IgG4-related disease and increased the likelihood of flare-free complete remission at 1 year, confirming the role of CD19-targeted B cell depletion as a potential future strategy for maintenance treatment in IgG4-related disease and also IRC. 113 The CD19-FC receptor 2B is another therapeutic target to inhibit B cells, plasmablasts, and CD19-expressing plasma cells using obexelimab and showed first promising results in a pilot phase 2 trial. 114 The clinical effects in IgG4-RD of belimumab targeting the BAFF are currently also under study.

Maintenance treatment of IgG4-RD should be patient-tailored based on predictors for relapse, comorbidities, and the risk of (developing) glucocorticosteroid-induced side effects. 33 In our clinic, the standard of care was during the last decade to induce remission in IRC with medium-dose prednisolone for (2 to) 4 weeks, after which prednisolone was tapered down and an immunomodulator (azathioprine, starting dose 50 mg daily; alternatively, 6-mercaptopurine, or mycophenolate mofetil) was added at mostly moderate doses. The optimal duration of maintenance therapy has not been established, but at least 2 to 3 years appear reasonable based on data available, and long-term treatment beyond 3 years may be warranted in individuals with a high risk of relapse including multiorgan IgG4-RD, markedly elevated serum IgG4, involvement of hilar and intrahepatic bile ducts in IRC, multiple strictures, or thicker bile duct walls. 21

A potential role for ursodeoxycholic acid (UDCA) in the maintenance treatment of IRC has not been studied so far. 3 33 Anticholestatic, hepato-, and cholangioprotective effects of UDCA have been shown for several fibrosing cholangiopathies including PBC and PSC, and its beneficial effect for overall transplant-free survival is meanwhile clearly documented for PBC. 115 Putative mechanisms of action of UDCA in fibrosing cholangiopathies have been intensely studied and discussed. 116 UDCA at adequate doses (∼15 mg/kg/day) might provide nonspecific bile duct protection without relevant side effects in addition to immunosuppressive treatment in IRC and might, thereby exert an additional glucocorticosteroid-sparing effect at the level of the biliary tree.

When relevant advanced fibrotic bile duct strictures in IRC do not adequately react (any longer) on glucocorticosteroid treatment, endoscopic intervention under antibiotic prophylaxis with balloon dilatation and—if unresponsive to balloon dilatation alone—short-term stenting may be needed to guarantee adequate bile passage into the duodenum. 33

Depending on the clinical course, patients with IRC are seen at a 6 to 12-month interval at the outpatient clinic to assess the development of potential biliary and liver damage, other organ involvement, risk of malignancy, and management of their therapy-induced side effects. Currently, life-long surveillance is advised for patients with IRC. 33 104

As IRC is associated with type I AIP in ≥90%, monitoring of exocrine and endocrine pancreatic function is recommended. Exocrine pancreatic insufficiency has been reported to occur in up to 53% of individuals with IRC and faecal elastase tests should be performed when indicated (e.g., steatorrhea and weight loss). 17 20 Endocrine pancreatic dysfunction leading to diabetes mellitus type 3c has been reported in one IRC cohort to occur in 37%, but may even be higher during the long-term course.

Biliary cirrhosis in IRC cohorts of the Mayo Clinic has been reported in 4.5% 117 —7.5% 21 and may be more frequent in people with proximal bile duct involvement (9%, 5 years). Annual transient elastography appears as an advisable strategy to monitor fibrosis progression in IRC in line with recommendations for PSC and PBC. 33 118 Management of IRC-related biliary cirrhosis should include semi-annual HCC screening and varices screening according to guidelines. Also, the risk of splanchnic and portal vein thrombosis in up to 9% of IRC patients should be considered 20 and treated accordingly.

The risk of malignancy in IRC has been reported variably in different Japanese cohorts. In one large cohort, 25/527 (4.7%) IRC patients developed malignancies during a follow-up of 4.1 years comparable to an age/gender-matched control population. 19 Other studies report an increased risk with 11 to 22% of patients experiencing a malignancy during their disease course 20 64 119 including a prominent increase in pancreatic and biliary tract malignancies. 64 119

Renal impairment in individuals with IRC occurs in up to 12%, especially in patients who have kidney, ureter, or retroperitoneal involvement of IgG4-RD, 20 and should be managed in collaboration with experienced nephrologists/urologists.

Patients treated with long-term glucocorticosteroids should be assessed for osteoporosis risk on a regular base and supplemented with calcium/vitamin D. Endocrine pancreatic function should be monitored by HbA1c on a regular basis, and exocrine pancreatic function by faeces elastase measurement.

## Conclusion

The insight into clinical and diagnostic aspects, pathophysiological mechanisms, and potential therapeutic approaches in IRC has remarkably evolved during the last two decades and will continue to grow thanks to the intense collaborative efforts of dedicated basic and clinical researchers in the field. Among the major unmet needs for the next years will be the development of novel biomarkers for accurate and fast diagnosis of IRC to distinguish IRC from other cholangiopathies such as PSC and CCA to avoid unnecessary and potentially invalidating surgical and medical interventions, a deeper insight into its pathophysiology and the continuing development of efficient targeted, well-tolerated and affordable therapies.
